# Increased susceptibility to repeated freeze-thaw cycles in *Escherichia coli *following long-term evolution in a benign environment

**DOI:** 10.1186/1471-2148-6-104

**Published:** 2006-12-05

**Authors:** Sean C Sleight, Nicholas S Wigginton, Richard E Lenski

**Affiliations:** 1 Department of Microbiology and Molecular Genetics, Michigan State University, East Lansing, MI 48824, USA; 2 Department of Geosciences, Virginia Polytechnic Institute and State University, Blacksburg, VA 24061, USA

## Abstract

**Background:**

In order to study the dynamics of evolutionary change, 12 populations of *E. coli *B were serially propagated for 20,000 generations in minimal glucose medium at constant 37°C. Correlated changes in various other traits have been previously associated with the improvement in competitive fitness in the selective environment. This study examines whether these evolved lines changed in their ability to tolerate the stresses of prolonged freezing and repeated freeze-thaw cycles during adaptation to a benign environment.

**Results:**

All 12 lines that evolved in the benign environment for 20,000 generations are more sensitive to freeze-thaw cycles than their ancestor. The evolved lines have an average mortality rate of 54% per daily cycle, compared to the ancestral rate of 34%. By contrast, there was no significant difference between the evolved lines and their ancestor in mortality during prolonged freezing. There was also some variability among the evolved lines in susceptibility to repeated freeze-thaw cycles. Those lines that had evolved higher competitive fitness in the minimal glucose medium at 37°C also had higher mortality during freeze-thaw cycles. This variability was not associated, however, with differences among lines in DNA repair functionality and mutability.

**Conclusion:**

The consistency of the evolutionary declines in freeze-thaw tolerance, the correlation between fitness in glucose medium at 37°C and mortality during freeze-thaw cycles, and the absence of greater declines in freeze-thaw survival among the hypermutable lines all indicate a trade-off between performance in minimal glucose medium at 37°C and the capacity to tolerate this stress. Analyses of the mutations that enhance fitness at 37°C may shed light on the physiological basis of this trade-off.

## Background

Most research in evolution pursues the comparative method, in which the present-day patterns of organismal diversity are examined in order to infer historical processes of change [1]. Research in paleontology allows a more direct examination of the past [2], but fossil data are limited in certain respects, including the inability to measure the performance abilities of organisms. A third approach for studying evolution is to perform long-term studies, either observational [3] or experimental [4], that allow one to observe evolution in action across many generations. In recent years, bacteria and viruses have become especially popular for experimental evolution, owing to their rapid generations that allow studies to run for hundreds or even thousands of generations [5-10].

In a long-term experiment, Lenski and colleagues have propagated 12 populations of *E. coli *for more than 20,000 generations at 37°C in a minimal-salts medium supplemented with glucose [5,11,12]. The dynamics of both phenotypic [13-19] and genomic [20-26] evolution have been characterized in a variety of ways. During the 20,000 generations, the bacteria have genetically adapted to their selective environment, such that their mean fitness relative to the ancestor increased by about 70%, based on direct competitions [15]. Interestingly, four of the 12 populations evolved defects in their DNA repair mechanisms, which caused them to become hypermutable [13,15]. The evolving bacteria also increasingly became ecological specialists, in the sense that their performance in some, but not all, other test environments tended to decline [14,15,17,26]. The parallel trajectories between increasing fitness in the selective environment and declining performance in other environments suggest that most of the decline in other environments is the result of pleiotropic side-effects of the same mutations that produce adaptation in the selective environment [15]. The fact that the four populations that became mutators do not show much more specialization is also consistent with this interpretation [15].

Stressful environments, such as prolonged freezing or repeated freeze-thaw cycles, may reveal other performance tradeoffs in the evolved lines. In fact, freezing and thawing impose several interconnected stresses including dehydration, hyperosmotic stress, ice formation, oxidative stress, and low temperature [27-29]. The acute responses of bacterial cells to freezing and thawing, including the effects of prior exposure to cold and other stresses on survival [30-36], are reasonably well understood. Many other variables also contribute to whether bacteria survive freezing and thawing, including their nutritional status and growth phase as well as the cooling rate employed [28,29,37]. Freezing and thawing *E. coli *cells without an exogenously supplied cryoprotective agent, such as glycerol, severely decreases their viability [38,39]. Loss of viability is proportional to the number of freeze-thaw cycles that cells experience [37,40,41]. Therefore, the elapsed time that cells are frozen generally influences viability less than the processes of freezing and thawing.

By contrast, much less is known about how and why different bacterial strains and species vary in their capacity to survive these stresses [28,35,42,43]. In this study, we examine how evolutionary adaptation by populations of *E. coli *to serial propagation on a minimal glucose medium at a constant temperature of 37°C affected survival during prolonged freezing and repeated freeze-thaw cycles in the absence of cryoprotectant. In particular, we test whether there was an evolutionary trade-off such that adaptation to this benign environment led to correlated losses in survival capacity under these stresses. We also evaluate whether replicate lines that evolved under the same regime show heterogeneous changes in their stress responses. One of our motivating interests in this research is to identify strains and conditions suitable for a future experiment that will investigate evolutionary adaptation to repeated freeze-thaw-growth cycles. We want to identify conditions in which some survival is possible, but where there is sufficient mortality to impose strong selection. Also, evidence for heritable variation among lines in survival under these extreme conditions would indicate the potential, at least, for evolutionary adaptation in that future experiment.

## Results and Discussion

### Effects of freeze-only and freeze-thaw regimes on survival of the ancestor

**Figure 1:**
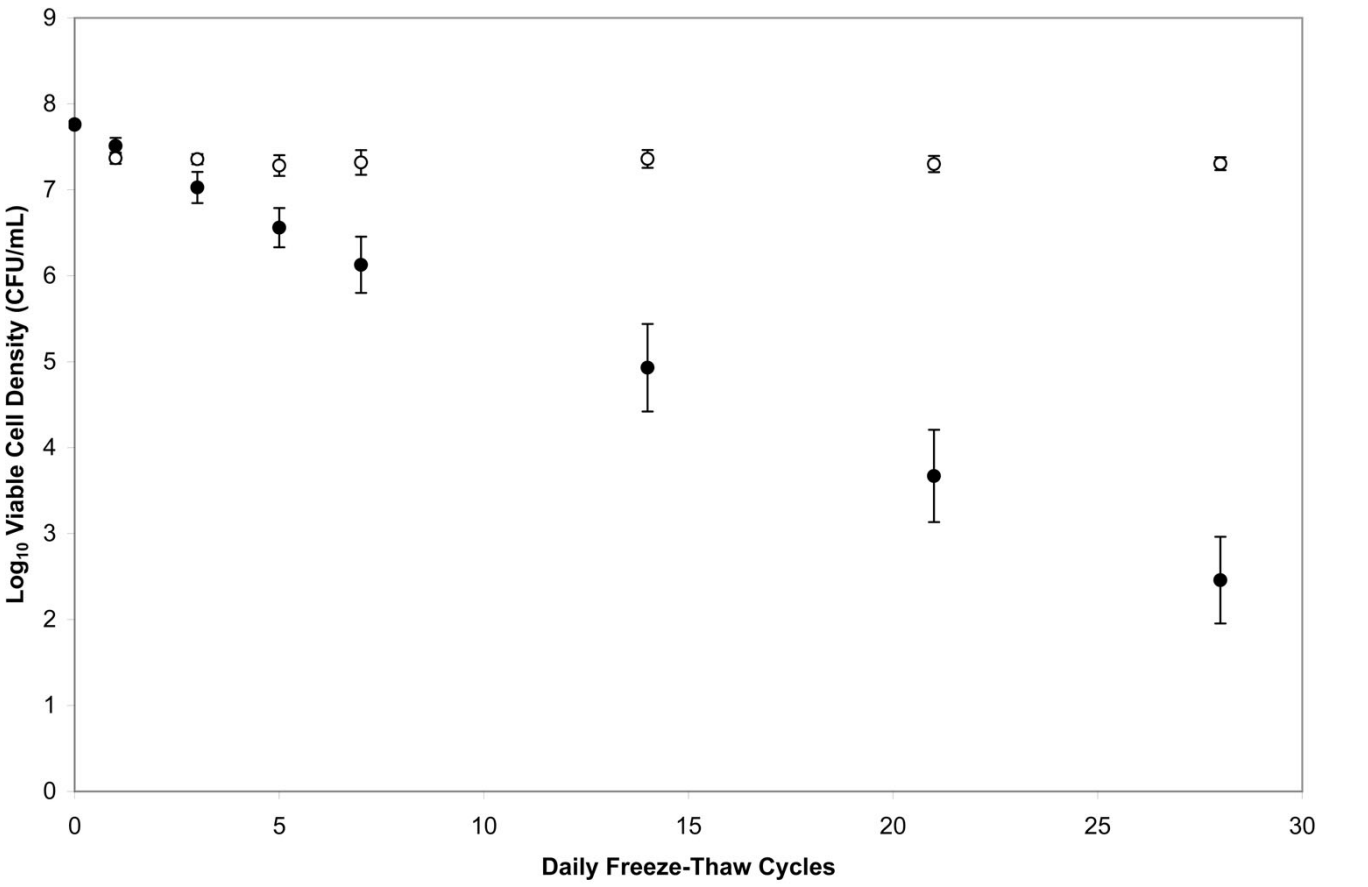
**Survival of ancestral *E. coli *strain under the freeze-only (prolonged freezing) and repeated freeze-thaw cycle regimes**. Each point is the log_10_-transformed viable cell density (CFU/mL) averaged over six replicates. Closed circles show the repeated freeze-thaw regime, and open circles show the prolonged freeze-only regime. Error bars are 95% confidence intervals and, when not visible, are smaller than the corresponding symbol.

Figure 1 shows the survival trajectories for the *E. coli *B ancestral strain under the -80°C freeze-only and freeze-thaw regimes over the course of 28 days. With daily freeze-thaw cycles, the density of viable cells declined by about five orders of magnitude. As evidenced by the log-linear trajectory (r^2 ^= 0.97, p < 0.0001), the bacteria experienced a nearly constant mortality rate of 34.4% killed per freeze-thaw cycle.

It is quite clear that most of this mortality was caused by the repeated bouts of freezing and thawing, as opposed to the time that cells spent frozen, because the cumulative mortality under the freeze-only regime was far less. In fact, over the entire 28 days at -80°C, with one thaw, the viable population size declined by only 35.2%. This decline almost exactly matches the cell death observed after one day in the freeze-thaw regime (Fig. 1), implying that no further death occurred during the other 27 days at constant -80°C. In fact, the slope of the cell-survival trajectory from day 1 to day 28 under the freeze-only regime was not significantly different from zero (p = 0.3483). Thus, there was little or no mortality, even over several weeks, beyond that caused by the single freeze-thaw cycle that was a necessary part of the survival assay procedures for all samples, regardless of how long they had spent at -80°C.

Therefore, the ancestral strain used for the long-term evolution experiment at 37°C is quite hardy with respect to prolonged freezing at -80°C. However, it is much more sensitive to repeated cycles of freezing and thawing. In the next section, we examine whether the lines that previously evolved for 20,000 generations in a benign environment became less tolerant of either prolonged freezing or repeated freeze-thaw cycles.

### Effects of freeze-only and freeze-thaw regimes on survival of the evolved lines in comparison with the ancestor

**Figure 2:**
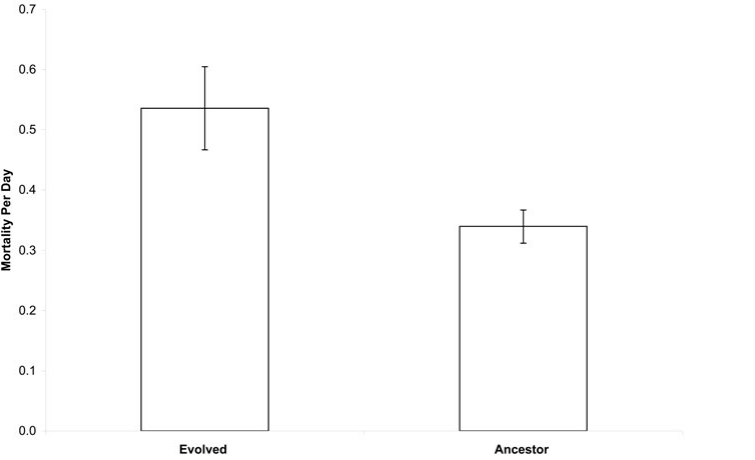
**Comparison of mortality rates between the evolved *E. coli *and their ancestor under the repeated freeze-thaw regime**. The height of each bar shows the mean mortality rate per day for the evolved lines or their ancestor, measured over ten daily freeze-thaw cycles. For the evolved bacteria, the mean is calculated over all 12 lines, with three assays for each line. For the ancestor, the mean is calculated over two marked variants (Ara^+ ^and Ara^-^), again with three assays for each one. See the Materials and Methods section for the mortality rate calculation. Error bars are 95% confidence intervals based on the number of lines (evolved) or total assays (ancestor).

We performed 10-day experiments under both freeze-only and freeze-thaw regimes using the 12 lines that evolved by serial propagation on a minimal glucose medium at constant 37°C for 20,000 generations and their ancestor. Each evolved line had three replicates, while the ancestor was replicated six-fold (three each for the Ara^- ^and Ara^+ ^marker variants). Figure 2 compares the average mortality rates of the evolved lines and their ancestor under the freeze-thaw regime. The ancestor experienced a mortality rate of 34.0% per day under the freeze-thaw regime, a value almost identical to our first experiment. By contrast, all 12 evolved lines experienced greater mortality, with an average rate of 53.7% per day. The difference in freeze-thaw mortality rates between ancestral and evolved rates is highly significant (two-tailed t-test with unequal variances, p < 0.0001).

**Figure 3:**
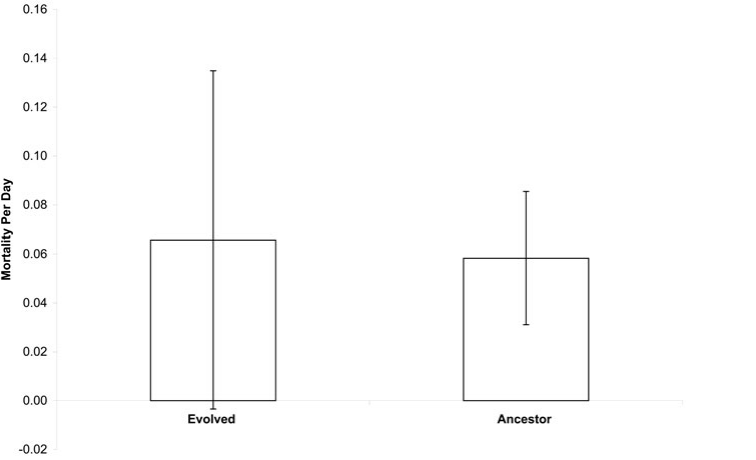
**Comparison of mortality rates between the evolved *E. coli *and their ancestor under the prolonged freeze-only regime**. The height of each bar shows the mean mortality rate per day for the evolved lines or their ancestor, measured over ten days at -80°C. For the evolved bacteria, the mean is calculated over all 12 lines, with three assays for each line. For the ancestor, the mean is calculated over two marked variants (Ara^+ ^and Ara^-^), with three assays for each one. See the Materials and Methods section for the mortality rate calculation, which includes an adjustment for the mortality caused by thawing in the final day. Error bars are 95% confidence intervals based on the number of lines (evolved) or total assays (ancestor).

However, we observed no significant difference between the evolved lines and their ancestor in mortality rates during prolonged freezing at -80°C (Fig. 3; two-tailed t-test with unequal variances, p = 0.7689). Mortality rates under this regime were calculated, as described in the Materials and Methods, such that they correct for the effect of one cycle of freezing and thawing. The average mortality rates estimated for the ancestor and evolved lines under the freeze-only regime were 5.8% and 6.6% per day, respectively. The ancestral value is somewhat higher than estimated in the first experiment, but it is still much lower than the mortality rates measured during repeated freeze-thaw cycles in either experiment.

Thus, the 37°C-evolved lines as a group remained about as robust as their ancestor to the effects of prolonged freezing at -80°C. However, the evolved lines are much more sensitive than their ancestor to the effects of repeated freeze-thaw cycles, with the average mortality rate increasing from 34.0% to 53.6% per daily cycle. In the section that follows, we examine variation among the evolved lines in their freeze-thaw sensitivity.

### Heterogeneity among the evolved lines in freeze-thaw survival

**Figure 4:**
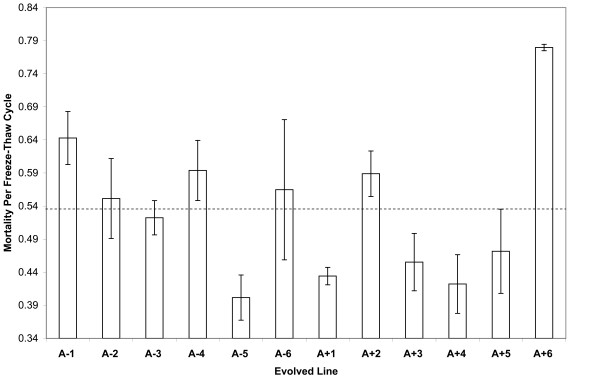
**Heterogeneity of mortality rates among the 12 evolved lines under the repeated freeze-thaw regime**. The height of each bar shows the mean mortality rate per day for one of the evolved lines, measured over ten daily freeze-thaw cycles, calculated from three assays for each line. The x-axis value (34%) is the estimated mortality rate of the ancestor; the dashed line shows the average mortality rate for the 12 evolved lines. Error bars are 95% confidence intervals calculated using the replicate assays for each line. See Table 1 for the statistical analysis testing for variation among the evolved lines.

**Figure 5:**
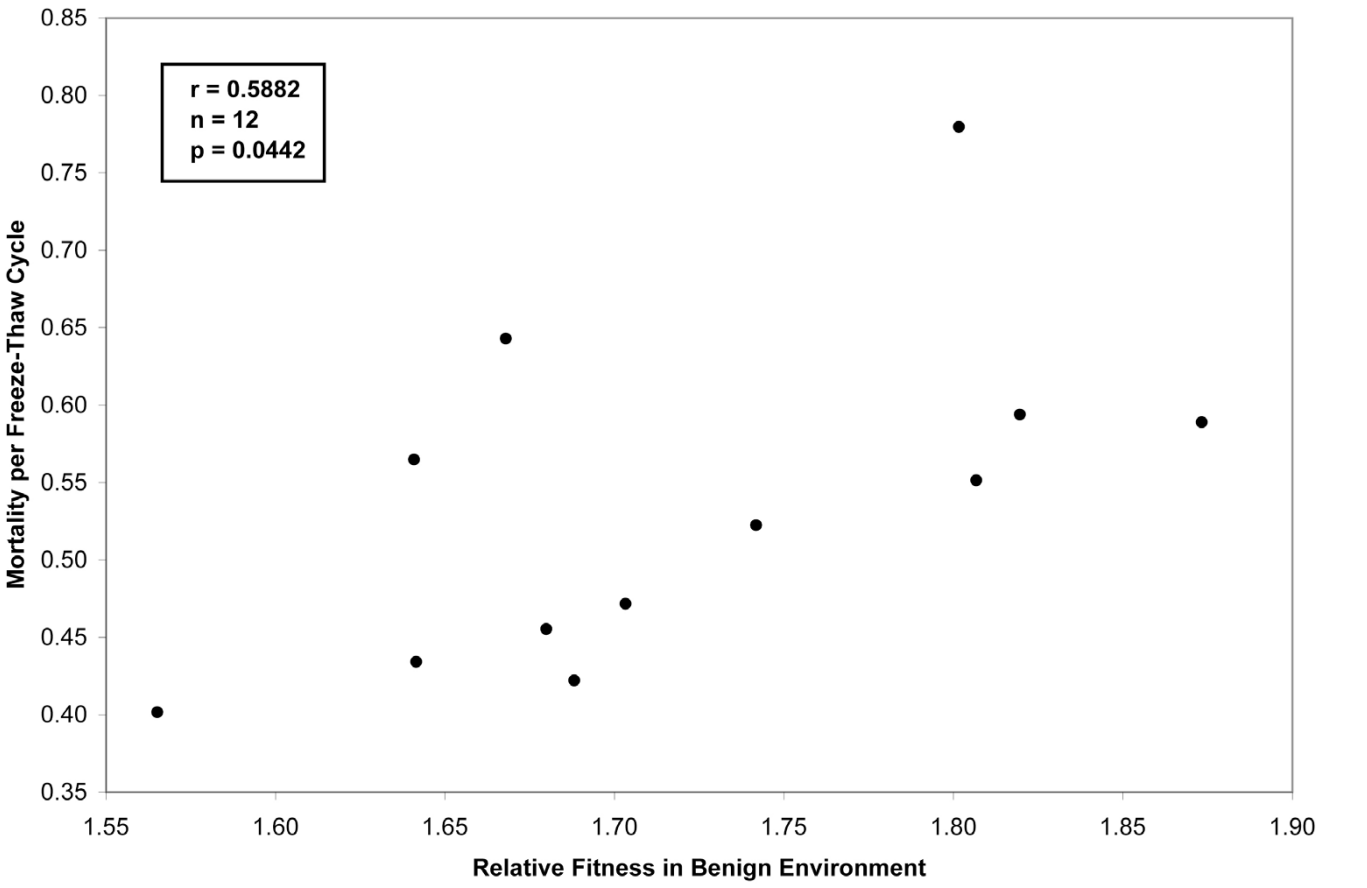
**Correlation between freeze-thaw mortality and fitness in the benign environment**. Each point shows the mortality rate per freeze-thaw cycle measured in this study, and the relative fitness measured previously [15] in the benign environment, for one of the 12 evolved lines.

Figure 4 shows the mortality rate per daily freeze-thaw cycle for each of the 12 lines that independently evolved at 37°C. The 95% confidence intervals were calculated by using the three replicate assays performed for each line. Estimated mortality rates vary from 40.2% to 78.0% per day. An analysis of variance confirms that variation among the evolved lines is highly significant (Table 1, p < 0.0001). Moreover, Figure 5 shows there is a significant correlation between competitive fitness measured in the benign selective environment [15] and mortality rates measured in the freeze-thaw regime (r = 0.5882, n = 12, two-tailed p = 0.0442). This correlation provides further support for the trade-off between fitness in the benign environment of serial propagation on glucose minimal medium at constant 37°C and survival during repeated freeze-thaw cycles.

**Table 1:** ANOVA testing for heterogeneity among the evolved lines in mortality rates under the repeated freeze-thaw regime.

**Source**	**df**	**SS**	**MS**	**F**	**p**
Line	11	0.3906	0.03551	88.67	<0.0001
Error	24	0.0096	0.00040		

Mortality rates were estimated with three-fold replication for each of the 12 evolved lines. The very low p-value indicates significant heritable variation among these lines in their survival capacity under the freeze-thaw regime.

The four lines that evolved defects in DNA repair, and which have much higher mutation rates (A-2, A-4, A+3, and A+6; refs. 13, 15), do not have consistently higher mortality rates, as a group, than do the other evolved lines. Also, there is no significant correlation between competitive fitness in the benign environment and survival under the freeze-only treatment (r = 0.0756, n = 12, two-tailed p = 0.8154), consistent with the absence of any significant difference between the ancestor and evolved lines as a group in this stress-related capacity.

The variation among lines that evolved in the benign selective environment indicate that they acquired different sets of mutations that differentially affect their sensitivity to freeze-thaw cycles. The differences between lines are not associated with variation in DNA repair function and mutability, which implies that pleiotropic effects of the mutations that were selected for their beneficial effects during the evolution experiment in minimal glucose medium at 37°C are responsible for the increased susceptibility to freezing and thawing. If, alternatively, mutations that harmed freeze-thaw survival had accumulated by neutral drift, then we would expect significantly higher susceptibility among the four lines with defective DNA repair functions [15,44].

## Conclusion

*E. coli *B cells experience little mortality during prolonged freezing at -80°C, even in the absence of added cryoprotectant. However, they are much more susceptible to repeated freezing and thawing, consistent with earlier studies [37,40,41]. Twelve lines that previously evolved for 20,000 generations in a benign environment, consisting of serial transfer in a minimal glucose medium at constant 37°C, all became more susceptible to freeze-thaw mortality than was their ancestor. Moreover, those evolved lines with higher fitness gains in the benign selective environment also tended to have greater susceptibility to freeze-thaw cycles, further supporting the trade-off in performance between these environments. However, the variation among the evolved lines was not associated with differences in DNA repair function and mutability that arose during the evolution experiment [13,15]. Therefore, increased susceptibility to freeze-thaw cycles in the evolved lines probably reflects pleiotropic effects of mutations that were beneficial to the bacteria during evolution in the benign environment with minimal glucose medium and constant 37°C. In any case, significant variation among the lines in their freeze-thaw survival implies that there is genetic variation for this trait, such that it can be selected, which will be a focus of our future research.

Future research directions include evolving *E. coli *populations under freeze-thaw-growth cycles. The growth phase will allow populations to recover from mortality caused by freezing and thawing; selection should favour a reduction in freeze-thaw mortality, a faster transition to growth after thawing, or both. Another future direction includes identifying individual mutations responsible for increased susceptibility to freezing and thawing in the lines evolved in the benign environment of constant 37°C, or increased resistance in lines evolved under the freeze-thaw-growth regime. Finally, phenotypic and genetic analyses of freeze-thaw resistance could be extended to natural isolates of *E. coli*. Comparisons between food-borne pathogens and commensals would be of particular interest because the ability to survive and recover from freezing and thawing might be an important adaptation of some food-borne pathogens.

## Methods

### Long-term evolution experiment and bacterial strains

The long-term evolution experiment is described in detail elsewhere [5,12]. In brief, 12 populations were founded using an *E. coli *B ancestor and then propagated at 37°C for 20,000 generations (3,000 days) in Davis minimal medium supplemented with glucose at 25 mg/L (DM25). The populations were diluted 100-fold daily into fresh medium, and their re-growth allowed about 6.6 (= log_2 _100) generations per day. The source strain, REL606, cannot grow on arabinose (Ara^-^), and it was used to start six populations; the other six were started with a spontaneous Ara^+ ^mutant, REL607, of the source strain. The Ara marker is selectively neutral in the experimental environment [5]. The 12 evolved lines used in this study were isolated as single clones at generation 20,000, after which they have been stored in glycerol at -80°C.

### Methods for measuring survival under freeze-only and freeze-thaw regimes

#### Culture media and experimental pre-conditioning

Bacteria used for testing were removed from storage in the freezer, inoculated into LB (Luria-Bertani) medium, and incubated for 24 h at 37°C. A culture was then diluted 1:10,000 into DM25 and incubated for 24 h at 37°C. A final 1:100 dilution was made into fresh DM25 and incubated for 24 h at 37°C, at which time cells were well into stationary phase, having exhausted the glucose in the first 8 h or so. Hence, all cells in the experiments were physiologically acclimated to the same environmental conditions before their survival was measured during repeated freeze-thaw cycles or during prolonged freezing.

#### Measuring cell density

Colony forming units (CFU) were used to estimate viable cell densities. A pilot study was performed to find a range of serial dilutions that would allow accurate cell counts as populations declined during repeated freeze-thaw cycles. For the evolved lines that were most sensitive, no dilution was made on the final day of the freeze-thaw experiment, so that no line fell below the detection limit of ~10 cells/mL. Diluted or undiluted cell cultures were plated on tetrazolium-arabinose (TA) indicator agar [5] and incubated at 37°C for 24 h before counting.

#### Freeze-thaw regime

After pre-conditioning, 1 mL of a stationary-phase culture was transferred into each of three replicate vials, which were then immediately placed in a -80°C freezer. Each day the tubes were kept frozen for 22.5 h and allowed to thaw at room temperature (about 22°C) for 1.5 h. Viable cell density was measured after the desired number of freeze-thaw cycles. For example, in order to measure survival after 1 day, the vials were frozen for 22.5 h and thawed for 1.5 h before measuring cell density. To measure survival after 7 days, the vials were subjected to seven freeze-thaw cycles and the cell density was measured after the last thaw.

#### Freeze-only regime

After pre-conditioning, 1 mL of a stationary-phase culture was transferred into each of three replicate vials and placed in a -80°C freezer. After the desired duration, a vial was thawed for 1.5 h and viable cell density measured. For example, to measure the freeze-only survival after 1 day, vials were frozen for 22.5 h and thawed for 1.5 h before measuring cell density. To measure the freeze-only survival after 7 days, vials were frozen for 166.5 h (= 7 days minus 1.5 h) and thawed for 1.5 h. Thus, the elapsed time at -80°C is varied experimentally, and only a single thaw occurs regardless of duration under this regime.

### Calculations and statistical methods

The first experiment was performed to measure and compare the survival of the ancestral strain under two different 28-day regimes, one with daily freeze-thaw cycles and the other representing the freeze-only regime. In each case, linear regression was performed on the log_10_-transformed viable cell densities to estimate the daily mortality rate and determine whether it differed significantly from zero. Data from all 28 days, including day 0, were used to calculate the mortality rate under the freeze-thaw regime, whereas that initial value was excluded when calculating the mortality rate during the freeze-only regime in order to adjust for effect of the single episode of thawing that was experienced in all subsequent days.

The second experiment compared the survival of the ancestral and evolved lines under the freeze-only and freeze-thaw regimes. It also allowed us to test for heterogeneity in survival rates among the 12 independently evolved lines. This experiment lasted 10 days; given the temporal constancy of the mortality rates observed in the first experiment, we estimated viable cell densities on days 0 and 10 only. For each assay, we calculated the percentage survival as *s *= *n*_10_/*n*_0_, where *n*_0 _and *n*_10 _denote initial and final densities, respectively. For the freeze-thaw regime, we then computed the mortality rate per day as *m*_*FT *_= 1 - *s*^1/10^. For the freeze-only regime, we took into account that there were nine days of sustained freezing and one freeze-thaw cycle. Therefore, the freeze-only mortality rate was calculated as *m*_*FO *_= 1 - (*s*/(1 - *m*_*FT*_))^1/9^. For example, if the mortality rate per freeze-thaw cycle were 30%, then one would expect *s *= 0.7 under the freeze-only regime even with perfect survival during the other nine days. If there were also 2% daily mortality during the constant freezing, then one would expect overall survival under the freeze-only regime to be *s *= 0.7 × (0.98)^9 ^≅ 0.58. Also, to preserve the statistical independence of the replicate *m*_*FO *_estimates, each one was calculated using a unique paired estimate of *m*_*FT*_.

For each mortality rate parameter, *m*_*FT *_or *m*_*FO*_, we compared the evolved lines with the ancestral strain as follows. We first computed the mean of the three replicate assays for each evolved line, and then we computed the grand mean and standard deviation from the individual means of the 12 independently evolved lines. For the ancestor, we computed the mean and standard deviation over the six assays (three each for the two marker states). We performed a two-tailed *t*-test with unequal variances, which allows for the fact that the evolved lines may have diverged from one another as well from their common ancestor. For the freeze-thaw mortality rates, we also tested for heterogeneity among the evolved lines by performing a one-way analysis of variance (ANOVA), with the three replicate assays per line providing statistical replication.

## Authors' contributions

SS performed the first set of experiments, while SS and NSW performed the second set of experiments. REL generated the evolved bacteria used in this study and designed the experiments. SS and REL performed the statistical analyses and jointly wrote the paper. All authors read and approved the final manuscript.
